# Plasma Exo-miRNAs Correlated with AD-Related Factors of Chinese Individuals Involved in Aβ Accumulation and Cognition Decline

**DOI:** 10.1007/s12035-022-03012-0

**Published:** 2022-08-30

**Authors:** Lifang Wang, Hefu Zhen, Yuzhe Sun, Shuang Rong, Benchao Li, Zhijie Song, Zhili Liu, Zhiming Li, Jiahong Ding, Huanming Yang, Xiuqing Zhang, Haixi Sun, Chao Nie

**Affiliations:** 1 BGI-Shenzhen, Shenzhen, 518083 China; 2 China National GeneBank, BGI-Shenzhen, Shenzhen, 518120 China; 3 Shenzhen Key Laboratory of Neurogenomics, BGI-Shenzhen, Shenzhen, 518083 China; 4 Department of Nutrition and Food Hygiene, School of Public Health, Medical College, Wuhan University of Science and Technology, Wuhan, China; 6 James D. Watson Institute of Genome Sciences, Hangzhou, 310058 China; 5 College of Life Sciences, University of Chinese Academy of Sciences, Beijing, 100049 China; 7 BGI-Beijing, Beijing, 102601 China

**Keywords:** Exosome-miRNA, AD-related factors, Aβ, Cognition

## Abstract

Numerous studies have investigated the risk factors of Alzheimer’s disease (AD); however, AD-risk factors related miRNAs were rarely reported. In this study, AD-risk factor related miRNAs of 105 Chinese individuals (45 AD patients and 60 cognitively normal controls) were investigated. The results showed that Hsa-miR-185-5p, Hsa-miR-20a-5p, and Hsa-miR-497-5p were related to AD and education, Hsa-miR-185-5p, Hsa-miR-181c-5p, Hsa-miR-664a-3p, Hsa-miR-27a-3p, Hsa-miR-451a, and Hsa-miR-320a were related to AD and depression. Target prediction of above miRNAs showed that these miRNAs were involved in the generation and clearance of amyloid-beta (Aβ), important molecules related to cognition, and disease-activated microglia response to AD. It is worth noting that Hsa-miR-185-5p was related to both education and depression, whose decreased expression pattern in AD patients was alleviated by education and enhanced by depression, and participates in Aβ generation and accumulation. Our results indicated that certain education and depression factors can contribute to AD progression by modulating miRNA expression, implying that preventive interventions might alter AD progression in Chinese patients.

**Supplementary Information:**

The online version contains supplementary material available at 10.1007/s12035-022-03012-0.

## Introduction 

Alzheimer’s disease (AD) is a neurodegenerative disease and causes most of patients with dementia over the world. During the slow progression of AD, the steady cognitive and functional decline are modulated by genetic and environmental protective and risk factors, including vascular, lifestyle-related, and psychological factors [1, 2]. Observational studies have revealed that statistically a third of the AD cases worldwide can be attributed to the following seven commonly modifiable hazard factors: midlife hypertension, depression, diabetes mellitus, midlife obesity, low education, physical inactivity, and smoking [1, 3–6]. Social integration and leisure activity have also been shown to protect against cognitive decline and dementia [7–10].

In fact, miRNA expression level could be influenced by lifestyle factors. The expressions of miRNAs such as miR-935 and miR-4772 in peripheral blood mononuclear cells have been reported to be downregulated in weight loss participants under energy-restricted treatment [11]. Studies have shown that diet, as a lifestyle factor, can change the miRNA expression profile in hepatocellular carcinomas [12, 13] and esophageal carcinoma [14]. However, whether AD-related lifestyle factors could change the miRNA expression profile of AD patients remains unreported.

Exosomes with diameters 40–160 nm (average 100 nm) [15] contain many cellular components including non-coding RNAs (ncRNAs), mostly miRNAs [16], and have the ability to pass through the blood–brain barrier (BBB) [17], making them a promising source of biomarkers to reflect the pathological process in the brain. In the central nervous system (CNS), 70% of the miRNAs released by human brain cells and possibly regulate the transcription of more than one-third of the genes [18, 19]. Differential miRNA expression in AD patients has been observed in exosomes from the plasma [20], serum [21], and cerebrospinal fluid [22]. Recent studies have demonstrated that exosome miRNA (exo-miRNA) profile can be potentially used for an early diagnosis of AD [23]. Whether miRNA expression profile from plasma exosome change according to AD-related lifestyle factors should be investigated necessarily and eagerly.

In this study, we evaluated the miRNA expression profile in the plasma exosome of Chinese patients with age- and gender-matched AD and normal individuals using next-generation sequencing data and found the correlations with AD-related factors. We found that AD-related factors, such as education and depression were correlated with changes in miRNA expression. More importantly, many targets of these differentially expressed miRNAs were reported to modulate Aβ generation and clearance, and/or important molecules involved in cognition, and disease-activated microglia response to AD.

## Material and Methods

### Sample Information and Ethics Statement

This study design included 109 Chinese participants of Han descent (age- and gender-matched 47 AD patients (ADs) and 62 individuals with normal cognition (normal controls (NCs)) aged 60 years and older and performed miRNA expression profile analysis related to lifestyle factors. The data for the study population on sociodemographic, lifestyle variables, and history of diseases were collected by the standardized questionnaire. According to the recommendation from the National Institute on Aging-Alzheimer’s Association workgroups [24], the patients with AD were screened using Mini-mental State Examination (MMSE) and then diagnosed by neurologists using a battery of tests including Clinical Dementia Rating (CDR), Geriatric Depression Scale (GDS) and Hachinski Ischemic Scale (HIS), and Computerized Tomography (CT) scans for the brain. NCs were determined by trained interviewers using the montreal cognitive assessment based on published cut-offs [25], the MMSE scores of NCs were converted based on montreal cognitive assessment (MoCA) scores according to a previous study [26], and the average MMSE scores were 12.87 ± 4.12 and 28.28 ± 1.95 for AD patients and NCs, respectively. Exosome miRNA data extracted from the blood samples from above 109 Chinses participants (47 ADs and 62 NCs) described previously were used for further sequencing analysis (Sun et al., 2021).

### Assessment of Baseline Characteristics

The potential influencing factors of Alzheimer’s disease were collected based on previous report [27], including education, obesity, smoking, hypertension, diabetes, depression, physical inactivity, and social contact. In addition, numerous epidemiological studies also demonstrated that drinking tea, alcohol consumption, and leisure activity was associated with risks of Alzheimer’s disease and dementia [28–30]. Thus, the above demographic information, lifestyle factors, and history of disease were collected in this study. Body mass index (BMI) was calculated as the ratio of body weight to the square of body height (kg/m^2^, < 18.5, 18.5–23.9, 24.0–27.9, ≥ 28.0). GDS was used to assess the depression of AD patients [31]. Individuals scoring 10 or higher in GDS were considered suffering from depression. The education levels were categorized as the no-educated (illiteracy) and educated (including elementary school, middle school, high school, and college). The tea, smoke, and alcohol consumption of individuals were categorized as Yes or No. Having leisure activity was defined as participating in at least one of following mental items: reading, using the internet, playing cards/mahjong, calligraphy, handicrafts, watching TV, and playing chess. The social connection questionnaire included the following questions based on the previous study with some details modified [9]: (1) working status; (2) living arrangement; (3) frequency of direct and remote contact with children; (4) frequency of contact with neighbors; (5) marital status; (6) frequency of attending social activities (such as group activities organized by the community, neighborhood, or village committee, the elderly community, university for the elderly, religious activities); (7) conjugal relations. Social support was determined based on the affinity and satisfaction from the aforementioned contacts. The following details were recorded: (1) working status was denoted as 1 when the individual was working (including farm work) or involved in volunteer and social work, whereas it was 0 for individuals who were retired or did no work; (2) living status was denoted as 1 for individuals living with family, or a live-in nurse, or in a nursing home), whereas it was denoted as 0 for individuals living alone; (3) frequency of direct and remote contact with children was denoted as 1 for individuals living with their children, had a close relationship with them, or with a contact frequency that was measurable in weeks), whereas it was denoted as 0 when the individual had an general or indifferent relationship with their children or a contact frequency that was measurable in months or years; (4) frequency of contact with neighbors was denoted as 1 for high frequency and 0 for medium or low frequency; (5) marital status was denoted as 1 for individuals in marriage (including remarriage), and as 0 for those not in marriage (including single, divorced, or widowed); (6) frequency of attending social activities was denoted as 1 for individuals where the frequency was measurable in terms of weeks, and as 0 for when the frequency was measurable in months, years, or never; (7) conjugal relation was denoted as 1 for individuals having intimate relations, and 0 for those with normal or indifferent relations. Overall, social connection was categorized based on of the status of the aforementioned contacts, “High” indicates at least four positive contacts, “Moderate” indicates two or three positive contacts, and “Poor” indicates 0 or 1 positive contact. Details of individual sample were listed in Supplemental Table 1.

**Table 1:** Baseline characters of the study population

Characteristics		NC (*n* = 62)	AD (*n* = 47)	*P*
Age, mean (SD), years		71.2 (5.6)	73.5 (7.2)	0.062
Female, n (%)		43 (69.4)	31 (66.0)	0.707
MMSE (SD)		28.28 (1.95)	12.87 (4.12)	< 0.001
BMI, kg/m2				< 0.003
Underweight (< 18.5)		3 (5.1)	13 (27.7)	
Normal weight (18.5–23.9)		34 (57.6)	26 (55.3)	
Overweight (24.0–27.9)		19 (32.2)	8 (17.0)	
Obese (≥ 28)		3 (5.1)	0 (0.0)	
Education				< 0.001
No education		15 (24.6)	46 (97.9)	
Elementary school		15 (24.6)	1 (2.1)	
Middle school		12 (19.7)	0 (0.0)	
High school		6 (9.8)	0 (0.0)	
college		13 (21.3)	0 (0.0)	
Current smoking		4 (6.5)	12 (25.5)	0.005
Alcohol consumption		9 (14.5)	7 (14.9)	0.956
Tea drinking		18 (29.0)	2 (4.3)	0.001
Hypertension		26 (42.6)	15 (31.9)	0.256
Depression		0 (0.0)	24 (51.1)	< 0.001
Social connection				0.001
	Poor	4 (6.5)	6 (12.8)	
	Moderate	23 (37.1)	31 (65.9)	
	Rich	35 (56.5)	10 (21.3)	
Leisure activity		49 (79.0)	2 (4.3)	< 0.001
Physical activity		60 (96.8)	43 (91.5)	0.476

Data is presented as number (proportion %). Missing data: none. Abbreviations: *NC*, normal cognition; *AD*, Alzheimer’s disease; *BMI*, body mass index. Chi-squared tests was used for statistical analysis of categoric variables by SPSS software. *MMSE*, mini-mental state examination

### Exosome miRNA Extraction

The exosome miRNA extraction was using commercial extraction kit and described previously [23]. Briefly, plasma samples, around 0.5 ml from participated individuals (AD and NC), were collected and stored at − 80 °C until use. Firstly, ExoQuick™ plasma prep and exosome precipitation kit (Cat No. EXOQ5TM-1-SBI, Palo Alto, USA) was used to isolate the exosomes from each 500 ul plasma sample. Secondly, miRNeasy Serum/Plasma Kit (Cat No. 217084, QIAGEN, Hilden, Germany) was used to extract the miRNAs from precipitated exosomes of each sample. Thirdly, Bioanalyzer system (Agilent, Santa Clara, US) and its matched Agilent small RNA kit was used to measure the quality of miRNAs (including RNA integrity number (RIN)) of each sample. Finally, Nano Drop 2000 (Thermo Scientific) was used to detect the concentration of miRNA. All above experiments were followed by the manufacturers’ instructions.

### miRNA Library Construction, Sequencing, and Alignment Pipeline

All sequencing methods were previously described [23]. Briefly, MGIEasy Small RNA library Prep Kit (MGI, Shenzhen, China) was used for preparing the small RNA sequencing library of each sample. The pooled library of each sample was loaded and sequenced by BGISEQ-500 (BGI) library, and 2.2 Gb raw data (reads) was obtained for each sample. Low quality reads of raw data were removed by trimming adaptor sequences. After cleaning process, 70–500 Mb per sample clean reads were mapped to the reference genome (hg19) using Bowtie 2 [32] with 1 mismatch. The matched reads were aligned to mature miRNAs in miRbase version 20 [33], and the perfectly matched miRNAs were counted for later analysis.

### Differential miRNA Expression Analyses

**Fig. 1:**
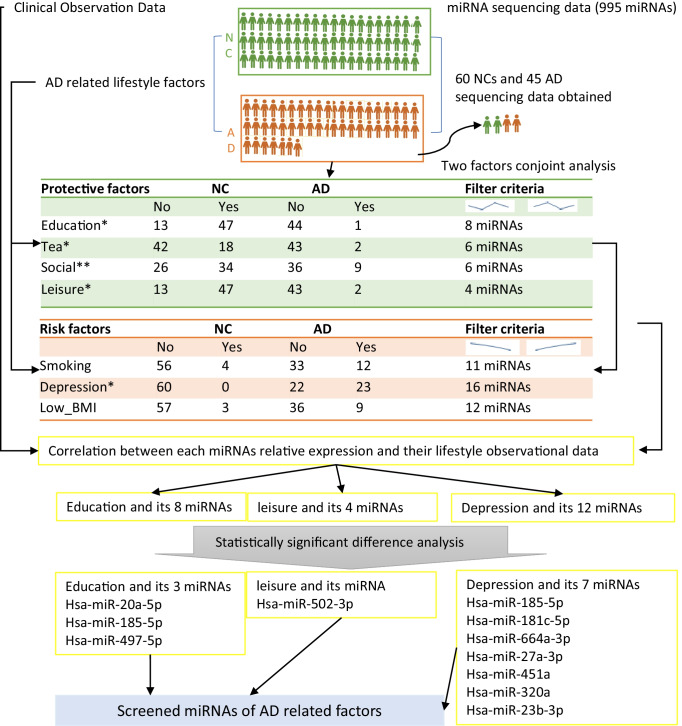
A schematic for the process of screening AD- and lifestyle-related miRNAs. (1) Protective and risk factors were statistically analyzed using clinical observational data. (2) Low quality miRNA sequencing samples were filtered, and AD- and lifestyle-related miRNAs were screened using conjoint analysis under filter criteria. (3) The lifestyles (education, leisure, depression), which miRNA expression data correlated to clinical observational data, were screened by canonical correlation analysis. (4) Expressed significantly different miRNAs related to AD and qualified lifestyles were selected for further target predictions. *The group with a few number of one group was leaving out for filtering. **The column “No” means “Low and mediate” and the column “Yes” means “High”

**Fig. 2:**
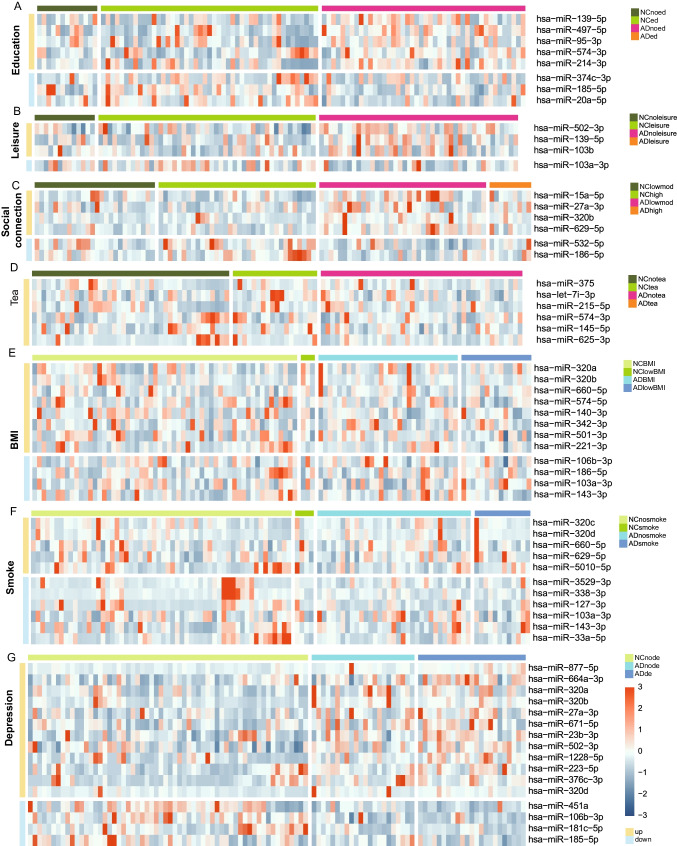
Heatmap of miRNAs for AD-related factors. **A**–**G**. Heatmap of miRNA expression level of all individuals with and without education (**A**), leisure activity (**B**), social connection (**C**), Tea drinking (**D**), BMI (**E**), smoke (**F**), and depression (**G**)

For protective factors such as education (Fig. 2A), differentially expressed miRNAs were identified based on fold change > 1.1 of the following ratios: NC-uneducated/NC-educated, and AD-uneducated/NC-uneducated; or NC-educated/NC-uneducated, AD-educated/AD-uneducated, and NC-uneducated/AD-uneducated (Fig. 1). The same criteria were used to identify miRNAs for leisure activities (Fig. 2B), social connection (Fig. 2C). AD-educated group and AD-leisure group were not considered this time because of few sample numbers (only one participant in AD-educated group, and only two participants in AD-leisure group) (Fig. 1).

For risk factors such as depression (Fig. 2G), miRNAs were also screened based on fold change > 1.1 of the following ratios: NC-no depression/NC-depression, AD-no depression/AD-depression, and NC-no depression/NC-depression; or NC-no depression/NC-depression, AD-no depression/AD-depression, NC-no depression/AD-no depression (Fig. 1). All above figures were performed by the Pheatmap package in R program.

### Statistical Analyses

In this population, 47 ADs and 62 NCs were performed the plasma miRNA analysis, and their basic characteristics were presented in Table 1 and Supplemental Table 1. Odd ratios (ORs) and 95% confidence intervals (95% CIs) were calculated for AD patients in Table 2 and Fig. 1. Canonical correlation analyses were performed to determine the correlations between lifestyle factors and expressed miRNAs specified in Table 3. The SPSS 24.0 was used for statistical analyses of the data presented in Tables 1 and 2. Prism 5.0 was used to perform one-way ANOVA analysis to determine the significant differences in miRNA expression for lifestyle factors such as education (Figs. 4B, E and 6A), leisure activity (Fig. 7B), and depression (Figs. 4F and I; 5A; 6D, G, and H; and 7A).

**Table 2:** Risk and protective factors of Alzheimer’s disease

Characteristics		Odd ratio (95% *CI*s)
		AD vs. NC
Underweight (< 18.5)		2.151 (1.509–3.066)
Yes		
No		
Education		0.028 (0.004–0.197)
Yes		
No		
Current smoking		1.993 (1.356–2.930)
Tea drinking		0.198 (0.052–0.748)
Alcohol consumption		1.017 (0.557–1.859)
Depression		3.696 (2.607–5.239)
Hypertension		0.766 (0.477–1.231)
Social connection		0.384 (0.214–0.690)
	High	
	low and moderate	
Leisure activity		0.038 (0.010–0.150)
Physical activity		0.626 (0.340–1.153)

**Table 3:** correlation analysis of specific expressed miRNA and lifestyle factors

	Canonical correlation analysis	*F* value	*P* value
Education	0.477	3.498	0.001
Smoke	0.386	1.476	0.154
Tea	0.261	1.194	0.316
Depression	0.605	3.170	< 0.001
BMI	0.221	0.713	0.661
Leisure activity	0.411	5.036	0.001
Social connection	0.300	1.963	0.091

Abbreviations: *BMI*, body mass index. Canonical correlation analyses were performed to determine any correlations between lifestyle factors and expressed miRNAs specified

### miRNA Target Prediction and Functional Enrichment Analyses

**Fig. 3:**
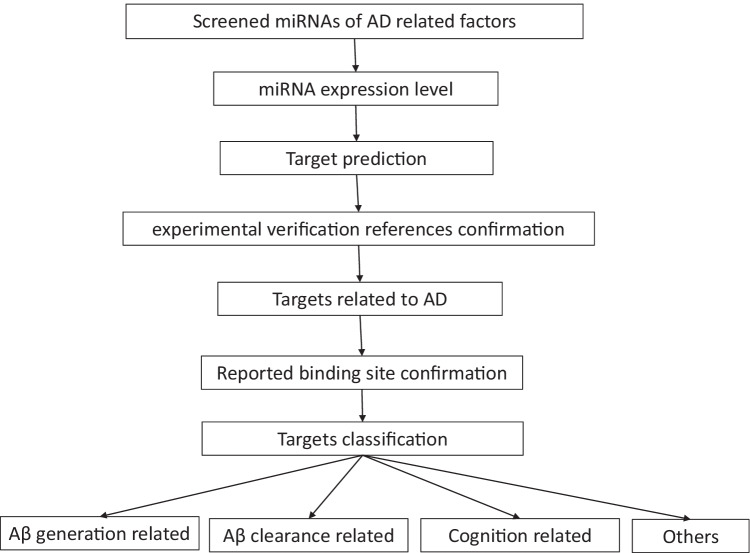
A roadmap for experimentally confirmed miRNA target prediction. (1) The expression levels for each miRNA were statistically confirmed. (2) Target predictions based on experimentally verified were preformed, and each target was confirmed upon searching reported research. (3) Targets related to AD were screened for further analysis. (4) Only reported binding site sequences of miRNA and mRNA were considered the miRNA’s target. (5) Qualified targets and miRNAs were classified to describe in this study

**Fig. 4:**
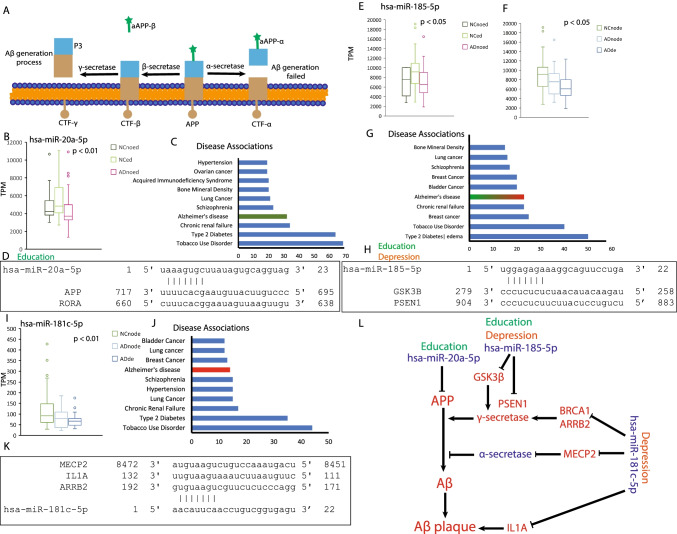
Education/depression and AD-related miRNAs were involved in Aβ generation. **A** A schematic of the catalytic enzyme in APP. **B** The relative expression level of hsa-miR-20a-5p categorized for education. NCnoed = normal controls who had no education experience, NCed = normal controls who were educated, ADnoed = AD patients who had no education experience, ADed = AD patients who were educated, *F*_(2, 102)_ = 5.455, *P* = 0.0056, one-way ANOVA followed by Tukey’s multiple comparison test. **C** Target enrichment analysis for hsa-miR-20a-5p. **D** Predicted binding site for hsa-miR-20a-5p and its targets. **E** The relative expression level of hsa-miR-185-5p categorized for education, *F*_(2, 102)_ = 4.071, *P* = 0.0199, one-way ANOVA followed by Tukey’s multiple comparison test. **F** The relative expression level of hsa-miR-185-5p categorized for depression, *F*_(2, 103)_ = 4.238, *P* = 0.0170, one-way ANOVA followed by Tukey’s multiple comparison test. NCnode = normal controls who did not suffer from depression, ADnode = AD patients who did not suffer from depression, ADde = AD patients who also suffered from depression. **G** The relative expression level of hsa-miR-185-5p. **H** Predicted binding site for hsa-miR-185-5p and its target. **I** The relative expression level of hsa-miR-181c-5p categorized for depression, *F*_(2, 103)_ = 5.441, *P* = 0.0057, one-way ANOVA followed by Tukey’s multiple comparison test. **J** Target enrichment analysis for hsa-miR-181c-5p. **K** Predicted binding site for hsa-miR-181c-5p and its targets. **L** A summary of miRNAs and their targets that are involved in Aβ generation process. Decreased expression levels of miRNA and genes were marked as diamond blue color, and increased expression levels of miRNA and genes were marked as red color. Factor education was using dark green color, and factor depression was using orange color

We predicted the targets of the miRNA using the R package multiMiR (V1.10.0). Validated targets in database were selected. Predicted cut-off and limit were set to 20 and 300, respectively. Functional enrichment analyses of target genes were performed using The Database for Annotation, Visualization, and Integrated Discovery (DAVID) v6.8 [34, 35] with default settings. Ten of the most enriched gene sets were selected for visualization (Figs. 4C, G, and J; 5B; and 6B and E) (Fig. 3). Mature miRNA sequences and the 3′ UTR of target mRNAs were downloaded from miRbase and USUC, respectively.

## Results

### Protective and Risk Factors of Alzheimer’s Disease

In order to investigate the protective and risk factors for Chinese AD patients, we analyzed the sociodemographic and lifestyle characteristics of 109 individuals. Our analysis revealed that 60 AD patients were significantly correlated with low BMI, low education level, depression, poor social connection, poor leisure activities, smoking habit, and low tea consumption (Table 1).

Further investigation of the protective and risk factors of AD was conducted by performing risk evaluation analysis, wherein the BMI categories “Normal weight,” “Overweight,” and “Obese” were merged into the category “No” in the “Underweight” category; education level categories “Elementary School,” “Middle School,” “High School,” and “College” were merged to the category “Yes” of Education; social connection categories “Low” and “Moderate” were merged into “Low and moderate” (Supplemental Table 1, Table 2). Our results show that the OR ratio (95% *CI*) for underweight was 2.151 (1.509–3.066), smoking was 1.993 (1.356–2.930), and depression was 3.696 (2.607–5.239), implying that these three factors were possible risk factors for Chinese AD patients. The OR ratio (95% *CI*) for education level was 0.028 (0.004–0.197), tea consumption was 0.198 (0.052–0.748), leisure activity was 0.038 (0.010–0.150), and social connection was 0.384 (0.214–0.690), implying that these factors were possible protective factors for Chinese AD patients (Table 2).

Therefore, higher education level, tea consumption, leisure activity, and high social connection were protective factors; whereas low BMI, smoking habit, and depression were risk factors for Chinese AD patients in this study.

### Exo-miRNA Expression Profile for AD-Related Factors

In order to determine whether miRNA sequencing results can provide insights to identify lifestyle factors involved in preventing AD, we screened the specific miRNAs related to above factors and AD by 45 ADs and 60 NCs from the previous sequencing data (2 ADs and 2 NCs were excluded for miRNA screening analysis because of low quality sequencing data). Of all miRNAs (995 miRNAs for each sample), 8, 4, 6, 6, 12, 11, and 16 miRNAs were possibly correlated with AD and education, leisure activity, social connection, regular tea consumption, BMI, habitual smoking, and depression respectively (Fig. 2). Except hsa-miR-451a that was reported to be related to depression [36, 37], all specific miRNAs were novel AD-associated-factor-related miRNAs. In order to further investigate the relationship between above factor-related miRNA profiles and the clinical measurement of these factors, we performed canonical correlation analysis, which revealed that miRNA expression was correlated with clinical measurements of education (*R* = 0.477, *P* = 0.01), depression (*R* = 0.605, *P* < 0.001), and leisure activity (*R* = 0.411, *P* = 0.001) (Table 2). Therefore, observational data for education, leisure activity, and depression were correlated with miRNA expression.

### hsa-miR-20a-5p, hsa-miR-185-5p, hsa-miR-181c-5p, and hsa-miR-502-3p Were Involved in Aβ Generation

Aβ was generated by the cleavage of amyloid protein precursor (APP) by β-secretase to form CTF-β (C-terminal fragment-beta), which was further cleaved by γ-secretase and then underwent several processes to form Aβ. α-secretase prevents the formation of Aβ by catalyzing APP to αAPP-α (alpha-cleaved APP- alpha) (Fig. 4A) [38, 39]. In order to analyze the function of these miRNAs, validated targets of significantly expressed miRNAs were predicted using DAVID. A decreasing pattern in AD was alleviated by education for hsa-miR-20a-5p (Fig. 4B) that targets several genes enriched in AD (Fig. 4C), including APP (Fig. 4D) [40], which is a well-known culprit of AD [41]. Decreased expression of hsa-miR-20a-5p increased APP expression, which further promoted Aβ generation, a significant signal for AD, which is a well-known culprit of AD and an experientially validated target of miR-20a-5p [41]. Decreased expression of hsa-miR-20a-5p may increase APP expression, which further promoted Aβ generation, a significant signal for AD.

Presenilin 1 (PSEN1), a catalytic component of the γ-secretase complex [42], plays a pivotal role in generating Aβ and increases its expression in AD patients. hsa-miR-185-5p was a decreasing miRNA in AD patients and the decreasing pattern was alleviated by education (Fig. 4E) and aggravated by depression (Fig. 4F). Functional enrichment analysis showed that validated targets were enriched in AD (Fig. 4G). One of these targets, PSEN1, contained the predicted binding site for hsa-miR-185-5p (Fig. 4H); the same was applied to GSK3B (glycogen synthase kinase 3 beta), another key player in AD pathology, which regulates APP and PSEN1 [43]. Decreased hsa-miR-185-5p in AD improves the expression of PSEN1 and GSK3B, which further increases Aβ generation.

Another decreased miRNA in AD patients was hsa-miR-181c-5p, whose decreasing pattern was aggravated by depression (Fig. 4I) and its targets were enriched in AD (Fig. 4J). Several targets of hsa-miR-181c-5p had binding sites in their 3′ UTRs (Fig. 4K). Breast cancer 1 (BRCA1), which has increased expression in AD patients, was predicted to be targeted by hsa-miR-181c-5p and reported to affect the turnover of PSEN1 (Fig. 4L) [44]. Methyl-CpG binding protein 2 (MeCP2), which was targeted by hsa-miR-181c-5p (Fig. 4K) and upregulated in AD patients, repressed α-secretase, ADAM metallopeptidase domain 10 (ADAM10), to improve Aβ generation (Fig. 4L) [45]. Arrestin beta 2 (ARRB2), upregulated in AD patients, as another target of hsa-miR-181c-5p, was reported to regulate γ-secretase [46]. IL1A was expressed in AD patients and directly related to Aβ generation [47], with a predicted binding site in 3′ UTR of hsa-miR-181c-5p (Fig. 4K).

Therefore, decreased expression of hsa-miR-20a-5p in AD patients increased APP expression and decreased hsa-miR-185-5p and hsa-miR-181c-5p, thereby increasing the expression of its targets, which further upregulates β-secretase and γ-secretase and downregulates α-secretase to increase Aβ generation and plaque formation. Education was the protective factor that alleviated Aβ generation, whereas depression exacerbated Aβ generation (Fig. 4L).

### hsa-miR-20a-5p, hsa-miR-181c-5p, and hsa-miR-664a-3p Were Involved in Cognitive Decline in Alzheimer’s Disease

**Fig. 5:**
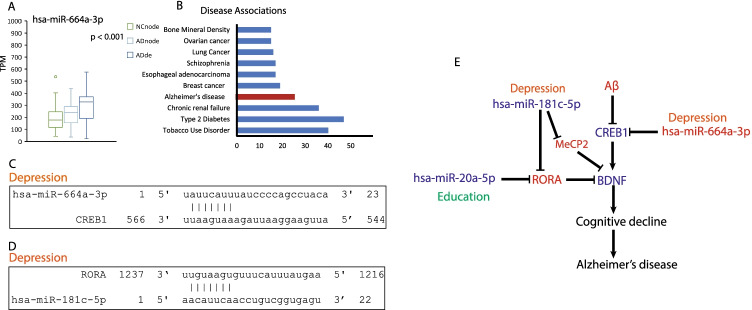
Education/depression- and AD-related miRNAs were involved in cognition decline. **A** The relative expression level of hsa-miR-664a-3p categorized for depression, *F*_(2, 103)_ = 8.439, *P* = 0.0004, one-way ANOVA followed by Tukey’s multiple comparison test. **B** Target enrichment analysis for hsa-miR-664a-3p. **C** Predicted binding site for hsa-miR-664a-3p and its target. **D** Predicted binding site for hsa-miR-181c-5p and its target. **E** A summary of miRNAs and their targets that are involved in cognitive decline in AD. Decreased expression levels of miRNA and genes were marked as diamond blue color, and increased expression levels of miRNA and genes were marked as red color. Factor education was using dark green color, and factor depression was using orange color

Brain-derived neurotrophic factor (BDNF) is well known as a beneficial marker for cognition; its deficit contributes to cognitive disorders and results in APP fragmentation [48]. Aβ reduces BDNF mainly by lowering phosphorylated cyclic adenosine monophosphate (cAMP) response element binding protein (CREB) [49]. hsa-miR-664a-3p has an increased expression in AD patients, and depression intensified its upregulation (Fig. 5A). CREB1 expression was inhibited by hsa-miR-664a-3p (Fig. 5B and C). RORA, a transcription factor that downregulates BDNF [50], was targeted by hsa-miR-181c-5p and hsa-miR-20a-5p that were correlated with depression and education, respectively (Figs. 4D and 5D). MeCP2 can also selectively repress the expression of BDNF [45]. A decrease in the expression pattern of these two miRNAs led to an increase in the expression of RORA, which in-turn inhibited BDNF expression in AD patients. Therefore, hsa-miR-664a-3p was upregulated in AD patients, which downregulated CREB1 and BDNF expression levels, thereby leading to a cognitive decline in AD patients. hsa-miR-181c-5p and hsa-miR-20a-5p were downregulated in AD patients and targeted both the transcription factors RORA that negatively regulated BDNF as well as MeCP2 whose upregulation further reduces the level of BDNF, thereby aggravating cognitive decline in AD patients (Fig. 5E).

### hsa-miR-451a, hsa-miR-27a-3p, hsa-miR-497-5p, hsa-miR-181c-5p, and hsa-miR-320a Were Involved in Aβ Clearance in Alzheimer’s Disease

**Fig. 6:**
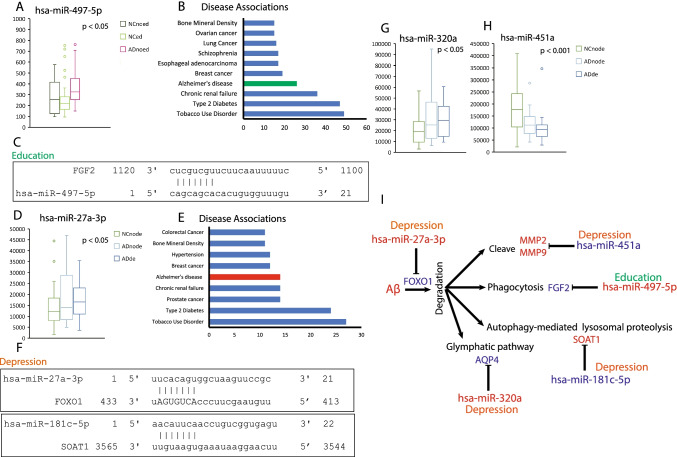
Education/depression- and AD-related miRNAs were involved in Aβ clearance. **A** The relative expression level of hsa-miR-497-5p categorized for education, *F*_(2, 102)_ = 4.791, *P* = 0.0103, one-way ANOVA followed by Tukey’s multiple comparison test. **B** Target enrichment analysis for hsa-miR-497-5p. **C** Predicted binding site for hsa-miR-497-5p and its target. **D** The relative expression level of hsa-miR-27a-3p categorized for depression, *F*_(2, 103)_ = 3.338, *P* = 0.0394, one-way ANOVA followed by Tukey’s multiple comparison test. **E** Target enrichment analysis for hsa-miR-27a-3p. **F** Predicted binding site for hsa-miR-27a-3p, or hsa-miR-181c-5p and their targets. **G** The relative expression level of hsa-miR-320a categorized for depression, *F*_(2, 103)_ = 4.605, *P* = 0.0121, one-way ANOVA followed by Tukey’s multiple comparison test. **H** The relative expression level of hsa-miR-451a categorized for depression, *F*_(2, 103)_ = 9.595, *P* = 0.0002, one-way ANOVA followed by Tukey’s multiple comparison test. **I** A summary of miRNAs and their targets that are involved in cognitive decline in AD. Decreased expression levels of miRNA and genes were marked as diamond blue color, and increased expression levels of miRNA and genes were marked as red color. Factor education was using dark green color, and factor depression was using orange color

We found that lifestyle- and AD-related miRNAs were also involved in Aβ clearance. Kiyota et al. (2011) injected fibroblast growth factor 2 (FGF2) into APP/PS1 mice to reveal that it significantly improved spatial learning and Aβ phagocytosis [51]. The expression of hsa-miR-497-5p was upregulated in AD patients and the upregulation pattern was inhibited by education (Fig. 6A). More than 20 targets of hsa-miR-497-5p were enriched in AD (Fig. 6B), and FGF2 level was regulated directly by hsa-miR-497-5p (Fig. 6C). Upregulated hsa-miR-497-5p in AD patients downregulated the expression of FGF2, thereby causing a decline in Aβ phagocytosis (Fig. 6I).

Forkhead box O1 (FOXO1), which promotes the clearance of Aβ, was reported to reduce the Aβ level in the cortices of AD mice [52]. The expression level of hsa-miR-27a-3p was increased in AD patients and depression exacerbated such an increase (Fig. 6D). More than 10 targets of hsa-miR-27a-3p were enriched in AD (Fig. 6E). Of the 10 targets, FOXO1 was directly bonded by hsa-miR-27a-3p (Fig. 6F). Upregulated hsa-miR-27a-3p in AD patients downregulated the expression of FGF2, which caused a decline in Aβ clearance (Fig. 6I).

Sterol O-acyltransferase 1 (SOAT1) inhibition has been reported to stimulate autophagy-mediated lysosomal proteolysis and increase Aβ clearance [53]. miRNA hsa-miR-181c-5p was downregulated in AD patients, and their downregulation pattern was aggregated by depression (Fig. 6I). Although there was no binding site in their 3′ UTRs, the two miRNAs were predicted to inhibit SOAT1 [54]. Downregulated hsa-miR-181c-5p caused an increase in SOAT1 expression, thereby inhibiting autophagy-mediated lysosomal proteolysis and Aβ clearance (Fig. 6I).

Aquaporin 4 (AQP4) is involved in Aβ clearance through the glymphatic pathway. AD led to a loss of polarity of AQP4 at the astrocytic endfeet, thereby causing more Aβ deposition because of a dysfunction in the glymphatic system [55]. AQP4 was predicted to be inhibited by hsa-miR-320a, which is increased by depression in AD patients (Fig. 6H). Reduced AQP4 possibly increased Aβ deposition owing to a dysfunction of the glymphatic system (Fig. 6I).

Metalloproteins, MMP2 and MMP9, have been reported to cleave soluble and fibrillar forms of Aβ [56]. MMP2 and MMP9 [57] were targeted by hsa-miR-451a, which was decreased by depression in AD patients (Fig. 6I). It is possible that an increase in MMP2 and MMP9 facilitates the cleavage of Aβ; however, it cannot keep up with the speed of Aβ accumulation in AD patients.

### Other miRNAs Related to AD-Related Factors

**Fig. 7:**
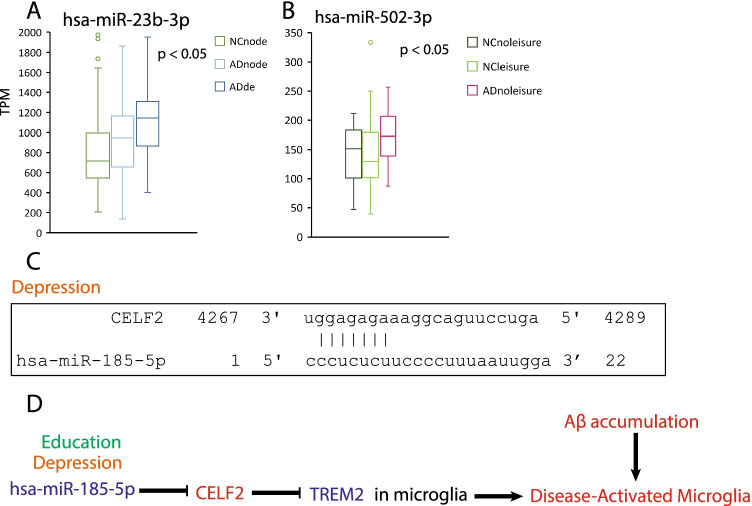
Other lifestyle- and AD-related miRNAs. **A** The relative expression level of hsa-miR-23b-3p categorized for depression, *F*_(2, 103)_ = 3.539, *P* = 0.0326, one-way ANOVA followed by Tukey’s multiple comparison test. **B** The relative expression level of hsa-miR-502-3p categorized for leisure activities, *F*_(2, 101)_ = 4.137, *P* = 0.0188, one-way ANOVA followed by Tukey’s multiple comparison test. NCnoleisure = normal controls who had no leisure activity, NC leisure = normal controls who had leisure activity, ADnoleisure = AD patients who had no leisure activity, ADleisure = AD patients who had leisure activity. **C** Predicted binding site for hsa-miR-185-5p and its target. **D** A summary of hsa-miR-185-5p and its targets that are involved in microglia response to Aβ accumulation. Decreased expression levels of miRNA and genes were marked as diamond blue color, and increased expression levels of miRNA and genes were marked as red color. Factor education was using dark green color, and factor depression was using orange color

There were two more miRNAs that were correlated with AD and lifestyle factors, even though no validated targets were identified so far. hsa-miR-23b-3p was upregulated in AD patients, and the upregulating pattern was enhanced by depression (Fig. 7A). hsa-miR-502-3p was also upregulated in AD patients; however, this expression pattern was alleviated by leisure activity (Fig. 7B). Loss of TREM2 (triggering receptor expressed on myeloid cells 2) function compromises the microglial response (the disease-activated microglia pattern) to the accumulation of Aβ [58]. Skipping the transcription of exon 3 of TREM2 causes nonsense-mediated mRNA decay, thereby causing a reduction in functional TREM2 in AD patients. CELF2 (CUGBP Elav-Like Family Member 2) has been linked to the exon3 transcription skipping, and its overexpression enhances TREM2 exon3 skipping [59]. hsa-miR-185-5p was downregulated in AD patients (Fig. 4E and F), which promoted CELF2 expression (Fig. 7C). Therefore, a decrease in the expression level of hsa-miR-185-5p in AD patients was enhanced by depression and reduced by education, thereby promoting its target CELF2, which reduced the TREM2 function in the microglia and further increased Aβ accumulation (Fig. 7D).

## Discussion

Our study found that several AD-related factors, including education, leisure-time mental activity, and depression were correlated to miRNA expression. Compared to NCs, a decreased expression pattern of hsa-miR-185-5p in ADs was enhanced by depression and alleviated by education significantly, and this miRNA was involved in Aβ generation (Fig. 4L) and accumulation (Fig. 7D). hsa-miR-181c-5p, which is a depression and AD-related miRNA (Fig. 4I), was involved in Aβ generation (Fig. 4L), cognitive decline (Fig. 5E), and Aβ clearance (Fig. 6I). hsa-miR-20a-5p, which was decreased in AD patients, was alleviated in people educated and involved in Aβ generation (Fig. 4L) and cognitive decline (Fig. 5E).

To our knowledge, this is the first report to correlate clinical observations with miRNAs expression profiles in AD and AD-related factors. In this study, our results provide new insight on how lifestyle- and AD-related miRNAs promote AD progression through validated targets. APP and PSEN1 are critical molecules for process of Aβ accumulation, the mainstream hypothesis for AD [38, 39]. APP was one target of hsa-miR-20a-5p, and reduced expression pattern of hsa-miR-20a-5p in AD patients improves APP expression, the subtract of Aβ. The decreasing expression pattern of hsa-miR-20a-5p in AD was alleviated in educated people. Presenilin 1 (PSEN1) is a catalytic component of the γ-secretase complex [42] and plays a pivotal role in generating Aβ and increases its expression in AD patients. PSEN1 is a target of hsa-miR-185-5p, a decreasing miRNA in AD patients. And its decreasing expression pattern was alleviated by education (Fig. 4E) and aggravated by depression (Fig. 4F). Besides, BDNF is a beneficial molecule for cognition and its expression level decreases in AD patient because of Aβ accumulation [49]. Several molecules influence BDNF expression directly including CREB1, MeCP2, and RORA, which were targeted by hsa-miR-664a-3p, hsa-miR-181c-5p, and hsa-miR-20a-5p (Fig. 5E). The expression pattern of above three miRNAs in AD patients caused BDNF deficiency through validated targets, and the miRNA expression trends were alleviated by education and aggregated by depression. Interestingly, the targets of our miRNAs are also enriched in various types of cancers. In fact, miRNAs participate in conducting and regulating many cellular processes such as proliferation, apoptosis, differentiation, metabolism, and immunity [60–65]. Abnormal miRNA expression is usually related to diseases such as cancers [66, 67], AD [68], and the coronavirus pandemic [69, 70]. Based on the miRNA expression panels, there is a hypothesis that AD and cancer (hematologic malignancies, colorectal, and lung cancer) are characterized by a shift in the expression of the same genes but in the opposite direction, which is supported by several reports [71–75]. Stánitz et al. [14, 76] reported lifestyle-related miRNA profile in gastric cancer and esophageal cancer samples. In gastric cancer samples, upregulated expression of miR-21 was related to low social status and habitual smoking, and the downregulation of miR-143 was related to habitual smoking. In esophageal cancer samples, increased expression of miR-205 was related to smoking, and reduced expression of miR-143 and miR-203 were related to low social status and smoking. Therefore, it is interesting to study the lifestyle factors involved in AD and cancer in future studies.

In addition, all the specific miRNAs related to AD-related factors, whose effects on the NCs are needed to be discussed. There is a report shows that miR-27a plays key roles in depression through regulating vascular endothelial growth factor A (VEGFA). Upregulated miR-27a is detected in hippocampal tissues and blood from rat with depression [77], which is consistent with our result that hsa-miR-27a is also upregulated in AD patients. However, the AD related factors, education and leisure time mental activities, whose miRNAs expression profile or mechanism have not been reported so far. Numerous reports revealed that leisure or social activity is effective in coping with depression, which suggested the correlation among these AD-related lifestyle factors [78], although no significant relation between miRNAs and social activity (Table 3). hsa-miR-27a-5p (Fig. 2) also suggested its importance in regulating the depression and Alzheimer’s disease, and hsa-miR-320b as well.

### Limitations

There were some limitations for the samples of miRNA expression used in this study, although our sample size (*n* = 105) did provide insights into AD-related factors. For example, only one person was educated and suffered from AD, only two AD patients have leisure activity, while no NCs suffered from depression (Table 1, Supplemental Table 1), these limitations indicate a scope for further study in this area. Moreover, although the validated targets of specific miRNA were predicted in this study, transcriptome or proteome would be used for validation and more information in our further research. Nevertheless, because of its novelty, there is no relative cohort from public database to validate our results. To another validated group from hospital needs quite a long time to collect enough sample size, but still another validated cohortshould be used for further validation for the specifically expressed miRNA and verifying the potential biomarkers for the AD-related lifestyle factors in the future. Thus, our next step is to search for cooperation from hospital and aging community and try to build a cohort with a relatively big sample size including more AD patients with education or with leisure activity. After gaining consistent data, we can focus on significant miRNAs’ function on AD-relative lifestyle factors and try to validate in animal model such as mouse and primates.

## Conclusion

In this study, we first reported AD and education/depression-related miRNAs. Specific expressed miRNAs were reported to be correlated with AD-related factors, such as hsa-miR-185-5p, hsa-miR-20a-5p, hsa-miR-181c-5p, and hsa-miR-497-5p. Besides, the predicted targets of these miRNAs were indicated to take part in Aβ generation and clearance, cognitive decline, and progression of AD. These findings provided clues of AD preventive interventions in Chinese people.

## Supplementary Information

Below is the link to the electronic supplementary material.

Supplementary file1 (XLSX 16 KB)

## Data Availability

The datasets used and/or analyzed during the current study are available from the corresponding author on reasonable request.
